# Supplementary material for: Interaction of amphiphilic lipoarabinomannan with host carrier lipoproteins in tuberculosis patients: Implications for blood-based diagnostics
Source: PLoS One. 2021 Apr 7;16(4):e0243337. doi: 10.1371/journal.pone.0243337 (PMC8026062; doi:10.1371/journal.pone.0243337)
Supplement: S1 File — (PDF) [file pone.0243337.s001.pdf]

Section S1

Selection of antibodies for assay development

In order to determine the optimal antibodies for use in the lipoprotein capture assay, we systematically assessed the performance of several antibodies that were available to us, including two from BEI Resources (monoclonal antibody CS40, anti-LAM polyclonal antibody (LAM-pab), and five different monoclonal antibody clones (24, 27, 29, 31 and 171) that were generously provided by the Foundation for Innovative New Diagnostics (FIND) (Figure S1) using a conventional sandwich ELISA. Although reagent intensive and associated with lower sensitivity for detection of LAM (LoD 300-500 nM)[15,22] compared to the waveguide platform[43], ELISA is a useful tool for the high-throughput screening of antibodies.

A typical ELISA experiment for the detection of LAM at various concentrations using FIND clones 24, 29 and 31 as the capture, and clone 171 as the reporter is shown in Fig S1. The limit of detection (LoD) for LAM using this assay was found to be 62.5 nM (assuming a molecular weight of 19 KDa for LAM) (n=3, per experimental condition) using clone 31 as the capture and clone 171 as the reporter. LoD was defined as 3 (s/n) where s/n is the standard deviation in observed measurements, and summary of s/n for the measurements is shown in Fig S1. Statistical significance was determined using one-way ANOVA with Fisher's least significant different test used for *post-hoc* analysis (**P<0.05). Antibodies were assessed for suitability for use in the lipoprotein capture assay based on three parameters (Table S1): a) stability (>6 weeks after labeling with either horse radish

peroxidase (HRP) or Alexa fluor 647, under refrigeration), b) specificity (no cross reactivity with *M. smegmatis* LAM), and c) sensitivity (use of <30 nM of antibody to obtain a limit of detection of ≤ 62.5 nM of LAM, which is the maximal sensitivity obtainable for this antigen by ELISA in our hands). Antibodies that did not satisfy all three criteria were not used in the waveguide-assays. For instance, the best sensitivity for detection of LAM using this method incorporated clone 31 as capture, and clone 171 as the reporter (Fig S1). However, clone 31 was unstable upon HRP labeling, and demonstrated cross reactivity with *M. smegmatis* LAM, and therefore, was not selected for further development. Using a similar assessment metric (Fig S1), FIND clones 171 and 24 were chosen for use in the lipoprotein capture assay.

S1 Table. Selection of monoclonal antibodies

Antibody	Source	Properties	Parameter		
			Stability	Specificity	Sensitivity
**CS40**	BEI	Monoclonal	***	***	**
**Pab**	BEI	Polyclonal	*	*	**
**24**	FIND	Monoclonal	***	***	***
**27**	FIND	Monoclonal	***	**	***
**29**	FIND	Monoclonal	***	*	**
**31**	FIND	Monoclonal	*	**	***
**171**	FIND	Monoclonal	***	***	***

Characterization, selection, labeling and preparation of antibodies

Five different FIND monoclonal antibodies (clones 24, 27, 29, 31 and 171) were evaluated for sensitivity and specificity of detection of LAM by Enzyme Linked Immunosorbent Assays (ELISA), and optimal antibodies were selected for further use. For ELISA screening, a sandwich assay protocol was used. The conditions described for this screening assay were shown to provide good sensitivity for detection from early screening measurements at various antibody concentrations using the BEI antibodies, and H37RV LAM. All the FIND antibodies evaluated in the screening ELISA were first labelled with Horse Radish Peroxidase (HRP) using the EZ-Link Plus Activated Peroxidase Kit from ThermoFisher.

Rabbit polyclonal anti-LAM antibody from BEI Resources was diluted 100-fold in carbonate buffer, and 100μl of this dilution was added to each well of a 96 well polystyrene ELISA plate, sealed, and incubated overnight at 4°C. Then, plates were washed thrice with PBS-0.5% Tween 20 (PBST, room temperature (RT), 2 min each), and PBS containing 2% BSA was added to each well as a blocking agent 2 hr, RT). After washing (2X, PBST and 2X, PBS), LAM (500nM), diluted fresh in PBS in silanized plastic tubes was added and incubated for 2 hrs at 37°C. Following wash, varying concentrations (20-150 nM each, based on the manufacturer's recommendation) of each of the reporter antibodies being evaluated were added and incubated for 2 hours at 37°C. It was determined that 100 nM of the reporter antibody offers maximal sensitivity of detection. Following the quantification of binding affinity and signal resolution with individual antibodies, combinations of antibodies were evaluated (all five, sets of 2, 3 and 4). From this antibody characterization, performance sensitivities were found to be even better using a cocktail of two antibodies, FIND clones 24 and 171.

With this choice of antibodies, cross-reactivity studies were performed to determine binding of these antibodies with H37RV LAM, ara-LAM from *M. smegmatis* and Lipomannan from *M. bovis*.

For use in the waveguide assay, the chosen reporter antibodies, FIND clones 24 and 171, were labeled using Alexa Fluor 647 fluorescent labeling kit from Life Technologies Inc, as per manufacturer's instructions. Each antibody was purified by gel filtration, and tested for binding activity (immunoblot). The concentration of the labeled antibody, and the degree of labeling were measured by ultra-violet visible (UV-Vis) spectroscopy. Three different batch aliquots were prepared and characterized. The degree of labeling for FIND 171 was 6, 7.6, 5.2 for each of these batches, and for FIND 24 was 7.5, 9.1 and 5.03. Aliquots of each antibody were prepared and stored at 4 °C. They were combined to make a cocktail of 15nM total antibody concentration before each experiment. Lipoprotein capture assay is a sandwich immunoassay, where the capture antibody targets the coat-protein of HDL nanodiscs, namely Apolipoprotein A1. This capture antibody, goat polyclonal anti-ApoA1 (1mg/ml), was purchased in biotinylated form and activity was measured by ELISA, and subsequently evaluated by immunoblot at periodic intervals (once every month). Aliquots of the capture antibody were stored at -20 °C, 100nM of the antibody being used as effective concentration in each experiment.

S2 Table: Patient demographics

Patient ID	Sex	Age (years)	CD4 cells/mm3	Infiltrates	Miliary infiltrates	Cavity	Urine LAM result
1	FEMALE	28	32				1
2	FEMALE	58	57	YES	NO	NO	1
3	FEMALE	39	1				1
4	MALE	31	5	YES	NO	NO	1
5	FEMALE	38	202				1
6	MALE	35	125	YES	NO	NO	1
7	MALE	35	32				1
8	FEMALE	25	25	YES	YES	NO	1
9	MALE	30	18	YES	NO	NO	1
10	MALE	45	230	YES	NO	NO	1
11	MALE	37	2				1
12	FEMALE	35	313	YES	NO	NO	0
13	FEMALE	27	59	YES	NO	NO	1
14	FEMALE	29	10	YES	YES	NO	1
15	MALE	43	10	YES	NO	NO	1
16	FEMALE	21	138	YES	NO	NO	1
17	FEMALE	38	27				1
18	MALE	25	14	YES	NO	NO	1
19	FEMALE	38	28	YES	YES	NO	1
20	MALE	30	61	YES	YES	NO	1
21	FEMALE	30	31	YES	YES	NO	0
22	FEMALE	.	305	YES	NO	YES	0
23	MALE	28	109	YES	NO	NO	0
24	FEMALE	21	199	YES	NO	NO	0
25	MALE	50	2	YES	NO	NO	0
26	FEMALE	32	348	YES	NO	NO	0
27	FEMALE	28	117	YES	NO	NO	0
28	FEMALE	34	7				0
29	FEMALE	29	616	YES	NO	YES	0
30	FEMALE	18	13	YES	NO	NO	0
31	FEMALE	48	56	YES	YES	NO	0
32	FEMALE	24	81	YES	NO	NO	0
33	MALE	33	14	YES	NO	NO	0

34	FEMALE	24	354				0
35	MALE	50	54	YES	NO	NO	0
36	FEMALE	37	67	YES	NO	NO	1
37	FEMALE	40	25				1
38	MALE	44	36	YES	YES	NO	0
39	FEMALE	29	.				
40	MALE	.	626	YES	NO	NO	1
41	FEMALE	19	19				0
42	FEMALE	29	635	YES	NO	NO	0
43	FEMALE	20	73				0
44	MALE	.	28	YES	NO	NO	0
45	MALE	.	206	NO	NO	NO	0
46	FEMALE	30	138	YES	NO	NO	0
47	FEMALE	28	36	YES	NO	NO	0
48	FEMALE	33	140	YES	NO	NO	0

. represents missing data, Urine LAM result- positive=1, negative=0
