# Supplementary material for: Interaction of amphiphilic lipoarabinomannan with host carrier lipoproteins in tuberculosis patients: Implications for blood-based diagnostics
Source: PLoS One. 2021 Apr 7;16(4):e0243337. doi: 10.1371/journal.pone.0243337 (PMC8026062; doi:10.1371/journal.pone.0243337)

**S1 Fig. Antibody screening and selection by colorimetric sandwich immunoassays.** Performance of antibody clones 24 (square), 27 (circle) and 31(triangle), using antibody 171 as the reporter, as assessed by sandwich colorimetric immunoassays is plotted as a function of concentration (n=3, per antibody, per concentration). Plot shows highest absorbance of clone 31, followed by 24, and finally, 27.


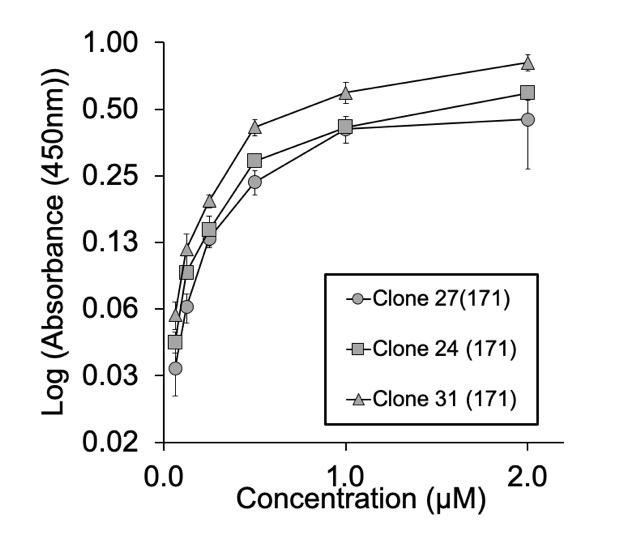

Supplement: S1 Fig — Performance of antibody clones 24 (square), 27 (circle) and 31(triangle), using antibody 171 as the reporter, as assessed by sandwich colorimetric immunoassays is plotted as a function of concentration (n = 3, per antibody, per concentration). Plot shows highest absorbance of clone 31, followed by 24, and finally, 27. (DOCX) [file pone.0243337.s002.docx]
