# Supplementary material for: Interaction of amphiphilic lipoarabinomannan with host carrier lipoproteins in tuberculosis patients: Implications for blood-based diagnostics
Source: PLoS One. 2021 Apr 7;16(4):e0243337. doi: 10.1371/journal.pone.0243337 (PMC8026062; doi:10.1371/journal.pone.0243337)
Supplement: Attachment — Submitted filename: Response to reviewers.docx [file pone.0243337.s003.docx]

**Editorial Comments:**

1. Please ensure that your manuscript meets PLOS ONE's style requirements, including those for file naming. The PLOS ONE style templates can be found at

https://journals.plos.org/plosone/s/file?id=wjVg/PLOSOne_formatting_sample_main_body.pdf and

https://journals.plos.org/plosone/s/file?id=ba62/PLOSOne_formatting_sample_title_authors*_affiliations.pdf*

***Response:*** *We confirm that the manuscript has been formatted according to the PLOS ONE requirements, as per the directions provided.*

2. Please provide additional details regarding participant consent. In the ethics statement in the Methods and online submission information, please ensure that you have specified (1) whether consent was informed and (2) what type you obtained (for instance, written or verbal, and if verbal, how it was documented and witnessed). If your study included minors, state whether you obtained consent from parents or guardians. If the need for consent was waived by the ethics committee, please include this information.

If you are reporting a retrospective study of medical records or archived samples, please ensure that you have discussed whether all data were fully anonymized before you accessed them and/or whether the IRB or ethics committee waived the requirement for informed consent. If patients provided informed written consent to have data from their medical records used in research, please include this information.
Once you have amended this/these statement(s) in the Methods section of the manuscript, please add the same text to the “Ethics Statement” field of the submission form (via “Edit Submission”).

For additional information about PLOS ONE ethical requirements for human subjects research, please refer to http://journals.plos.org/plosone/s/submission-guidelines#loc-human-subjects-research.

***Response:*** *The Ethics Statement in the manuscript has been modified and now reads as follows: “This study was approved by ethics committees of Johns Hopkins University School of Medicine, the Joint Clinical Research Centre (Kampala, Uganda), and Los Alamos National Laboratories. All participants provided written informed consent”.*

3. Thank you for stating the following in the Competing Interests section:

"I have read the journal's policy and the authors of this manuscript have the following competing interests: [insert competing interests here]"

We note that one or more of the authors are employed by a commercial company: Bako Diagnostics.

***Response:*** *The work was done by the researcher, Rama Sakamuri, when he was a post-doctoral fellow at LANL, with Harshini Mukundan. He has since moved to Bako Diagnostics. Since the affiliation of the agency where he worked for this project was LANL, we have edited this to say – “Los Alamos National Laboratory, Now at Bako Diagnostics”. Bako diagnostics did not participate or contribute to this work in any way.*

3.2. Please also provide an updated Competing Interests Statement declaring this commercial affiliation along with any other relevant declarations relating to employment, consultancy, patents, products in development, or marketed products, etc.

Within your Competing Interests Statement, please confirm that this commercial affiliation does not alter your adherence to all PLOS ONE policies on sharing data and materials by including the following statement: "This does not alter our adherence to  PLOS ONE policies on sharing data and materials.” (as detailed online in our guide for authors [http://journals.plos.org/plosone/s/competing-interests](about:blank)) . If this adherence statement is not accurate and  there are restrictions on sharing of data and/or materials, please state these. Please note that we cannot proceed with consideration of your article until this information has been declared.

***Response:*** *Based on the above comment, there are no competing interests and the company was not involved in the work in any way. Thus, there is no change in the competing interests. However, the competing interests statement has been revised and now reads as follows:*

**“Competing Financial Interests:** Scientists from the Los Alamos National Laboratories, operated by the Triad LLC, that are authors on this manuscript, do not have competing interests, and are not consultants for any competing interests.

Ramamurthy Sakamuri was a post-doctoral researcher at Los Alamos National Laboratory when he conducted this research. He has subsequently transitioned to Bako Diagnostics, which is his current affiliation. However, Bako Diagnostics did not participate or were not involved in the research presented in this manuscript in any way. This does not alter our adherence to PLOS ONE policies on sharing data and materials.”

Please include both an updated Funding Statement and Competing Interests Statement in your cover letter. We will change the online submission form on your behalf.

***Response:*** *We have included an updated funding statement and competing interests statement in the cover letter of the revised manuscript.*

*The funding statement now reads as follows:*

*“***Funding statement**: This work was supported by Los Alamos LDRD Directed Research Grant (Co-PI Mukundan) and grant R21 AI03599 to Dr. Susan Dorman from the National Institutes of Health. Los Alamos National Laboratory, an affirmative action equal opportunity employer, is managed by Triad National Security, LLC for the U.S. Department of Energy’s NNSA, under contract 89233218CNA000001. The funder provided support in the form of salaries for authors [HM, SJ and RMS from LDRD; SD and HM from NIAID], but did not have any additional role in the study design, data collection and analysis, decision to publish, or preparation of the manuscript. The specific roles of authors are articulated in the ‘author contributions’ section.”

Please know it is PLOS ONE policy for corresponding authors to declare, on behalf of all authors, all potential competing interests for the purposes of transparency. PLOS defines a competing interest as anything that interferes with, or could reasonably be perceived as interfering with, the full and objective presentation, peer review, editorial decision-making, or publication of research or non-research articles submitted to one of the journals. Competing interests can be financial or non-financial, professional, or personal. Competing interests can arise in relationship to an organization or another person. Please follow this link to our website for more details on competing interests: [http://journals.plos.org/plosone/s/competing-interests](about:blank)

4. Your ethics statement should only appear in the Methods section of your manuscript. If your ethics statement is written in any section besides the Methods, please move it to the Methods section and delete it from any other section. Please ensure that your ethics statement is included in your manuscript, as the ethics statement entered into the online submission form will not be published alongside your manuscript.

**Response: Ethics section has been moved to methods section**

5. Please include your tables only as part of your main manuscript and remove the individual files. Please note that supplementary tables should be uploaded as separate "supporting information" files.

**Response: Table 1 has been included in main manuscript and supplemental tables S1 and S2 are included in separate supplemental file**

6. Please include captions for your Supporting Information files at the end of your manuscript, and update any in-text citations to match accordingly. Please see our Supporting Information guidelines for more information: [http://journals.plos.org/plosone/s/supporting-information](about:blank).

**Response: Captions for supporting information files have been included at the end of manuscript.**

**Reviewer #1**:

1. *The study presents two methods for detection of lipoarabinomannan (LAM) in serum, as a tool to diagnose tuberculosis. Two methods were evaluated, the lipoprotein capture assay and the membrane insertion assay, both intended to solve the important problem with interaction of the amphiphilic LAM with host carrier lipoproteins. A very small number of serum samples are used to evaluate the two methods. It would be valuable to know more about the individual samples, and also about the possible use of these methods in clinical practice.*

**Response:** We have now included a table (Table S2) which captures more information on the samples in the revised manuscript. Specifically, gender, age, CD4 cells/mm3, identification of infiltrates and miliary infiltrates, cavity and urine LAM diagnostics (where available) have been included.

Additionally, we used existing de-identified serum specimens from six Ugandan adults who did not have TB symptoms and were not known to be HIV-positive; no additional meta-data were available for these specimens. Included in manuscript on page 9, lines 160-163.

2. The patient samples were selected from existing stored specimens that previously had been obtained for a study that evaluated the diagnostic accuracy of the Alere Determine™ TB LAM Ag assay (ref 27). In that study 1013 HIV-positive participants were enrolled. Among culture-positive tuberculosis patients, the Alere test identified 136/367 (37.1%) overall and 116/196 (59.2%) in the group with CD4 ≤100 cells per cubic millimeter.

   From these specimens, serum from 35 verified HIV positive TB cases, and 13 individuals with TB symptoms but no positive cultures, and 6 HIV negative controls were selected for this study. It would be important to know 1) the CD4 status of the 35 culture-confirmed TB patients and the 13 individuals with TB symptoms that were selected for inclusion in this study. The fact that the 6 control samples were from HIV-negative individuals while the 13 individuals with TB symptoms were HIV positive makes this information even more important

***Response:*** *The CD4 status of the patients has now been included in Table S2 of the revised supplemental document and a discussion of this has been included on Page 9, Lines 160-163 of the revised manuscript.*

3. It would also be important to know whether the samples were selected with knowledge of their Alere results.

***Response:*** *Samples were selected on the basis of culture results, and sent to LANL in a double blinded fashion. The Alere results were not taken into consideration for sample selection. Included on page 9, lines 159-160 of revised manuscript in clinical specimens section.*

4. The samples were too few to evaluate the sensitivity and specificity of the tests, especially the membrane insertion assay: only 8 samples were used to evaluate the membrane insertion assay, in parallel with the lipoprotein capture assay, and there were only 6 control samples, all negative in both assays. Based on the comparison between the two assays in the 8 samples the authors conclude that the membrane insertion assay was the most sensitive one.

***Response:*** *We agree with the reviewer that a much larger cohort should be analyzed to determine performance metrics in the clinical samples. This study was focused on establishing and optimizing the methods for more sensitive detection of serum LAM, and validating efficacy in clinical samples. To this end, the optimization of the study for both membrane insertion and lipoprotein capture (Figure 2b and Figure 3b) was performed in serum spiked with varying concentrations of LAM. The enhanced sensitivity of the membrane insertion assay over lipoprotein capture is noted in these spiked samples for known concentrations of the antigen. This trend is replicated in the small subset of clinical samples that we evaluated. This has been clarified in page 24, lines 481-486 of the revised manuscript.*

It would also be important to know how the authors envisage how the methods could be developed for use in clinical practice, since they include sophisticated instruments in both methods as well as harsh treatments (chloroform:methanol extraction) in the membrane insertion assay. The need to release LAM from host lipoprotein matrix is at the core of most work on development of methods to detect and quantify LAM in patient fluids, and crude chloroform:methanol extraction poses several problems.

***Response:*** *Our team has been working on resolving the engineering and simplification challenges for use of this technology at the point of need. We have developed and validated the first microfluidics device which is capable of performing lipid extractions within minutes at the point of need (Manuscript accepted with minor revisions pending to Nature scientific reports,  U.S. Provisional Patent Application No. 63/113,310). In addition, we have simplified the waveguide-based optical biosensor for field deployment, and validated assay performance on this new portable (<10Lbs) instrument with optical components integrated and the software for functionality on a phone-based application. This manuscript is in preparation, provisional patent application No  S133706.000, Entitled “Microfluidics Device”). We have added a generic description of these developments in lines 331-337, page 17 of the revised manuscript.*

5. From this point of view the lipoprotein capture assay seems more interesting, albeit apparently less effective, probably due to LAM epitopes being hidden by the lipoprotein matrix, a fact that should be discussed.

***Response:*** *We agree with the reviewer that both of these assays have relatively different advantages and disadvantages. With the developments in progress, as outlined in response to the previous question, we anticipate the membrane insertion assay will also become more field friendly. However, for the purpose of this manuscript, we have included a more detailed description of the advantages and disadvantages of the two methods, while briefly mentioning the developments in progress. These changes have now been included on page 15, lines 303-304, page 17, lines 331-337, page 6- lines 99-104 of the revised manuscript.*

The increased sensitivity of the membrane insertion assay over lipoprotein capture assay, even in spiked samples, indicates some of the LAM associated with lipoproteins is not readily detected. We agree with this assessment and have clarified this in the discussion on page 24, lines 486-488 of the revised manuscript.

6. Minor comment: Reference 17 is redundant and should be excluded or replaced by more recent references.

***Response****: This reference has been removed in the revised manuscript.*

**Reviewer #2**: The manuscript “Interaction of Amphiphilic Lipoarabinomannan with Host Carrier Lipoproteins in Tuberculosis Patients: Implications for Blood-based Diagnostics” by Mukundan et al. proposes two novel sandwich immunoassays for the detection of lipoarabinomannan derived from *Mycobacterium tuberculosis* in the blood. The assays are comprised by a capture element and a labeling element. The capture elements leverage LAM association with lipid particles or lipophilic phase and encompass: 1) lipid bilayer and 2) anti high-density lipoprotein antibody associated to a lipid bilayer. Labeling element uses an anti-LAM antibody, clone CS40.
The paper is well written and clearly presented. It has intellectual merit because it aims to bring additional evidence that LAM exists in the blood of tuberculosis patients in association with lipoproteins. The labeling antibody CS-40 mAb is well characterized in the literature and showed epitope specificity (DOI: https://doi.org/10.4049/jimmunol.1701673). Data present in the supplementary information support the choice of this antibody in comparison to other clones.

1. However, the design of the assay lacks rigor. The main issue is that “specificity controls were performed using IgG labelled with AF-647, rather than anti-LAM antibodies”. In order to ensure valid readings, immunoassay controls must be run using all the reagents used for the unknown samples (https://www.ncbi.nlm.nih.gov/books/NBK92434/). In the proposed assay it is especially crucial that the anti-LAM antibody is used for specificity controls, because it is the only element of the assay that confers specificity. Furthermore, the epitope of the IgG used for the negative control readings is not specified.

***Response:*** *We apologize if our original manuscript was not clear on the methods used.*

a) *Whereas CS40, anti-LAM antibody procured from BEI, was also evaluated, our initial assessments revealed that clones 171 and 24 from FIND performed best in our assay, as indicated in supplemental figure S1. Thus, all the developments described in the manuscript and the clinical evaluations were performed using a combination of FIND clones 171 and 24.*
b) *All specificity controls were also performed using FIND 171 and 24 as shown in supplemental figure S1, and not using a generic IgG labeled with AF-647. Thus, every single non-specific measurement reported in the manuscript was performed using the exact combination of anti-LAM antibodies as the specific measurements, and under exactly the same conditions of the assay. We have clarified these points in the methods section of the revised manuscript, on page 14, lines 280-281.*

2. On a minor note, the advantages of using an anti-HDL antibody linked to a lipid bilayer versus a less complicate alternative, such as ELISA plate coating, are not elaborated.

***Response:*** *We have elaborated on the advantages of the lipoprotein capture and membrane insertion assays, as compared to a conventional plate-based ELISA more clearly in the revised manuscript on page 7, lines 108-121. There are two limitations that impact the use of conventional plate-based methods for the detection of LAM. 1) LAM is an amphiphile and therefore, sticks to plastics and other surfaces, reducing assay reliability, sensitivity and reproducibility. 2) In clinical samples, LAM is already sequestered by host lipoproteins and not available in free-form(1). It has to be detected in this conformation or liberated from it to facilitate detection. In this manuscript, we have attempted to develop methods that address both these limitations. Both the assays are performed on a surface functionalized with lipid bilayer, and therefore override the challenges of using plastic ELISA plates in concert with amphiphiles.* *Further, we have consistently shown that the use of the waveguide platform offers at least 10X greater sensitivity than conventional plate-based ELISA, which is an advantage when it comes to sensitive detection of pathogen antigens in complex clinical samples(2,3).*

3. Limit of detection is usually presented as a general characteristic of the assay and not in function of the concentration of one sample (DOI: https://doi.org/10.4049/jimmunol.1701673). In the results section, the limit of detection of assay number one is not presented, and the limit of detection of assay number two is 8.5 nM, which for LAM corresponds roughly to 400 ng/mL. Discussion on the expected concentration range of LAM in serum is lacking. Limit Since S/N values are reported for patients, S/N curves for different concentration of spiked control serum samples should be presented (Fig. 2b, 3b).

***Response:*** *We have clarified the quantification of the assays more rigorously in the revised manuscript.*

*The concentration of LAM in patient serum has not been clearly established. Brock et al. showed a range of 0-132 pg/ml in individuals without HIV infection(4). Other studies have “extracted” LAM from serum, but direct measurement of the antigen has not been largely reported(5–7).*

*According to equation 2, LOD for lipoprotein capture assay is 69 nM, which corresponds to a concentration of 1900ng/mL of LAM. LOD for membrane insertion assay is 3.3 nM, which corresponds to a concentration of 114 ng/mL LAM. This clearly demonstrates the greater sensitivity of the membrane insertion assay over the lipoprotein capture assay in standard curves based on spiked samples. At 500 nM of LAM, the S/N by lipoprotein capture assay is 8.2, whereas that by membrane insertion assay is 782.*

*Because of the significant difference in fluorescence units between lipoprotein capture and membrane insertion for some of the patients, we have chosen to present the data in terms of S/N. However, we have included the conversion metrics above clearly in the revised manuscript to ensure the interpretation of the data is clear to the reviewer and the reader.*

4. Figure 5b reports discrepant values obtained from the two assays in individual samples, but a strategy to reconcile the values to obtain a quantitative assay is missing.
   The abstract should indicate that the assays are sandwich assays and use anti-LAM CS-40 mAb as a labeling antibody.

***Response:*** *Figure 5b represents the difference in sensitivity for the same clinical sample when two different methods are used to measure the antigen. One method is more sensitive than the other, and is responsible for the disparity in the signature intensity noted. We have expanded on this, and clarified the results, in the results section more clearly (page 22, line 440-442).*

*The antibody combination used in the assay was FIND Clones 171 and 24, and this has been clarified in the legends and in the methods as well.*

5. Demographic and clinical information of patients should be included in the manuscript if available.

***Response:*** *We have now included Table S2 in the revised manuscript, which includes meta-data with this information for the patients in the study.*

References:

1.	Sakamuri RM, Price DN, Lee M, Cho SN, Barry CE, Via LE, et al. Association of lipoarabinomannan with high density lipoprotein in blood: Implications for diagnostics. Tuberculosis. 2013;93(3):301–7.

2.	Mukundan H, Kubicek JZ, Holt A, Shively JE, Martinez JS, Grace K, et al. Planar optical waveguide-based biosensor for the quantitative detection of tumor markers. Sensors Actuators, B Chem. 2009;138(2):453–60.

3.	Kubicek-Sutherland JZ, Vu DM, Mendez HM, Jakhar S, Mukundan H. Detection of lipid and amphiphilic biomarkers for disease diagnostics. Biosensors. 2017;7(3):25.

4.	Brock M, Hanlon D, Zhao M, Pollock NR. Detection of mycobacterial lipoarabinomannan in serum for diagnosis of active tuberculosis. Diagn Microbiol Infect Dis. 2020;

5.	Sada E, Aguilar D, Torres M, Herrera T. Detection of lipoarabinomannan as a diagnostic test for tuberculosis. J Clin Microbiol. 1992;30(9):2415–2418.

6.	Crawford AC, Laurentius LB, Mulvihill TS, Granger JH, Spencer JS, Chatterjee D, et al. Detection of the tuberculosis antigenic marker mannose-capped lipoarabinomannan in pretreated serum by surface-enhanced Raman scattering. Analyst [Internet]. 2017;142(1):186–96. Available from: http://xlink.rsc.org/?DOI=C6AN02110G

7.	Owens NA, Young CC, Laurentius LB, De P, Chatterjee D, Porter MD. Detection of the tuberculosis biomarker mannose-capped lipoarabinomannan in human serum: Impact of sample pretreatment with perchloric acid. Anal Chim Acta. 2019;1046:140–7.
